# Bromodomain and extra‐terminal protein mimic JQ1 decreases inflammation in human vascular endothelial cells: Implications for pulmonary arterial hypertension

**DOI:** 10.1111/resp.12872

**Published:** 2016-08-18

**Authors:** Sharon Mumby, Natalia Gambaryan, Chao Meng, Frederic Perros, Marc Humbert, S. John Wort, Ian M. Adcock

**Affiliations:** ^1^ Vascular BiologyImperial College LondonLondonUK; ^2^ Airway Disease Section, National Heart and Lung InstituteImperial College LondonLondonUK; ^3^ Faculty of MedicineSouth Paris UniversityClamartFrance; ^4^ Pulmonary Hypertension: Pathophysiology and Therapeutic InnovationINSERM Research Unit 999ClamartFrance; ^5^ Pulmonary Resuscitation Respiratory and Service, National Reference Centre for Pulmonary Hypertension Severe, Assistance Publique Hôpitaux de ParisHôpital Antoine BéclèreParisFrance

**Keywords:** bromodomain and extra‐terminal proteins, human pulmonary microvascular endothelial cells, inflammation, proliferation, pulmonary hypertension

## Abstract

**Background and objective:**

Nuclear factor kappa B (NF‐kB)‐mediated inflammatory gene expression and vascular endothelial cell proliferation/remodelling are implicated in the pathophysiology of the fatal disease, pulmonary arterial hypertension (PAH). Bromodomain and extra‐terminal (BET) proteins are essential for the expression of a subset of NF‐kB‐induced inflammatory genes. BET mimics including JQ1+ prevent binding of BETs to acetylated histones and down‐regulate the expression of selected genes.

**Methods:**

The effects of JQ1+ on the proliferation of primary human pulmonary microvascular endothelial cells (HPMECs) from healthy subjects were measured by bromodeoxyuridine (BrdU) incorporation. Cell cycle progression was assessed by flow cytometry; mRNA and protein levels of cyclin‐dependent kinases (CDKs), inhibitors and cytokines were determined by reverse transcription‐quantitative PCR (RT‐qPCR), Western blotting or ELISA. Histone acetyltransferase (HAT) and deacetylase (HDAC) activities were determined in nuclear extracts from whole lung of PAH and control patients.

**Results:**

JQ1+ significantly inhibited IL6 and IL8 (IL6 and CXCL8) mRNA and protein in HPMECs compared with its inactive enantiomer JQ1−. JQ1+ decreased NF‐kB p65 recruitment to native IL6 and IL8 promoters. JQ1+ showed a concentration‐dependent decrease in HPMEC proliferation compared with JQ1−‐treated cells. JQ1+ induced G1 cell cycle arrest by increasing the expression of the CDK inhibitors (CDKN) 1A (p21^cip^) and CDKN2D (p19^INK4D^) and decreasing that of CDK2, CDK4 and CDK6. JQ1+ also inhibited serum‐stimulated migration of HPMECs. Finally, HAT activity was significantly increased in the lung of PAH patients.

**Conclusion:**

Inhibition of BETs in primary HPMECs decreases inflammation and remodelling. BET proteins could be a target for future therapies for PAH.

## Abstract 2

Endothelial cell inflammation and proliferation are important in the pathogenesis of pulmonary arterial hypertension (PAH). Bromodomain and extra‐terminal (BET) mimics reduced inflammation and cell proliferation in primary human pulmonary vascular endothelial cells. The enhanced histone acetyltransferase (HAT) activity in PAH suggests that BET mimics may be effective in PAH although experiments in disease cells/tissues are required.

**Abbreviations**
ANOVA: analysis of variance
ATS/ERS: American Thoracic Society / European Respiratory Society
AUC: area under the curve
BET: bromodomain and extra‐terminal
BRD: bromodomain‐containing protein
BrdU: bromodeoxyuridine
CDK: cyclin‐dependent kinase
CDKN: cyclin‐dependent kinase inhibitor
ChIP: chromatin immunoprecipitation
COPD: chronic obstructive pulmonary disease
CXCL8: IL 8 (protein)
DAPI: 4′,6-diamidino-2-phenylindole
EGF: epidermal growth factor
EGM2: Endothelial growth medium 2
ELISA: enzyme‐linked immunosorbent assay
ET‐1: endothelin‐1
FACS: Fluorescence-activated cell sorting
FCS: foetal calf serum
FGF: fibroblast growth factor
HAT: histone acetyltransferase
HDAC: histone deacetylase
HPMEC: human pulmonary microvascular endothelial cell
Ig: immunoglobulin
IL: interleukin 6
IPAH: idiopathic PAH
LPS: Lipopolysaccharide
MCP‐1: Monocyte chemoattractant protein 1
MMLV: Moloney-murine leukemia virus
MTT: 3‐(4,5‐dimethyl‐2 thiazolyl)‐2‐5‐diphenyltetrazolium bromide
NF‐kB: nuclear factor kappa B
PA: pulmonary artery
PAH: pulmonary arterial hypertension
PASMC: PA smooth muscle cell
PDGF: Platelet-derived growth factor
RT‐qPCR: reverse transcription‐quantitative polymerase chain reaction
RV: right ventricle

## INTRODUCTION

Pulmonary arterial hypertension (PAH) describes a group of diseases characterized by raised pulmonary vascular resistance, resulting from vascular remodelling in the pre‐capillary resistance arterioles.^1^ Patients die from right heart failure within 3 years of diagnosis if untreated. Treatment with vasodilators, such as endothelin (ET) receptor antagonists, prostacyclin analogues and phophodiesterase type V inhibitors, has improved both morbidity and mortality^2^; however, these are not a cure and the mortality rate in patients is still unacceptably high.^2^ It is, therefore, important to understand the mechanisms of vascular remodelling in PAH and to determine novel therapies targeting these abnormalities.

The endothelial cell plays a key role in vascular remodelling. Early on in the disease process, increased apoptosis within the endothelial layer leads to survival of cells with an apoptosis‐resistant phenotype, resulting in proliferation, which is a hallmark of later disease.^3^ We and others have shown that pulmonary vascular endothelial cells in patients with idiopathic PAH (IPAH) demonstrate markers of increased inflammation, such as nuclear factor kappa B (NF‐kB) activation.^4^ Moreover, in an in vitro condition, endothelial cells produce cytokines that are also found circulating in the plasma of patients with IPAH and correlate with worse survival.^5^

Histone acetylation, under the control of histone acetyltransferases (HATs), is linked to heightened inflammatory gene expression.^6^, ^7^ Acetylated lysine residues are removed by histone deacetylases (HDACs).^8^ Acetylated histones are recognized by bromodomain and extra‐terminal (BET) proteins to enable co‐ordinated regulation of genes involved in cell proliferation, apoptosis and inflammation.^9^, ^10^ Bromodomain‐containing protein (BRD) 4 was significantly increased in PAH lung tissue, distal pulmonary arteries (PAs) and right ventricle (RV) compared with control tissues. This over‐expression was also demonstrated in isolated PAH PA smooth muscle cells (PASMCs) compared with control cells. BRD2 and BRD3 were increased in the distal PAs from PAH patients compared with controls, although the difference seen in the BRD3 levels was not significant. In the RV, there was no increase in BRD2 and only a small increase in BRD3 was observed.^11^ Additionally, BRD4 expression was induced during RV hypertrophy in a rat PAH model.^12^ The BET proteins BRD2 and BRD4 are involved in inflammatory gene expression in several murine and human cell types^13^, ^14^, ^15^, ^16^ and their binding to acetylated histones can be blocked by molecular mimics such as JQ1+ resulting in attenuated cell proliferation and differentiation in vivo and in cell lines.^13^, ^17^ In an in vivo condition, BRD4 inhibition using JQ1 or siBRD4 reversed established PAH in the Sugen/hypoxia rat model and decreased proliferation, increased apoptosis and restored mitochondrial membrane potential in PAH‐PASMCs suggesting that BRD4 up‐regulation may be pathologically associated with PAH in animal models.^11^ I‐BET151, a structurally similar BET mimic, reduces inflammation in in vivo and in vitro conditions following LPS challenge.^13^

The aims of this study were first to investigate a role for BET proteins (using JQ1+) in the regulation of inflammation and markers of vascular remodelling in human pulmonary microvascular endothelial cells (HPMECs). Second, to determine the HAT:HDAC ratio in PAH and control lung tissue to evaluate any potential differences in acetylation status within the lung environment.

## METHODS

### Subjects

Control specimens (*n* = 10) were obtained from lung resected during lobectomy for a solitary pulmonary nodule. Specimens from patients with IPAH (*n* = 10) were obtained following lung transplantation. The study was approved by the local ethics committee and all patients gave informed consent (Comité de Protection des Personnes Ile‐de‐France, Paris VII). Two to four randomly selected blocks of lung tissue (size 2 × 2.5 cm) were taken from the sub‐pleural parenchyma of the lobe obtained at the time of surgery, avoiding areas of tumour in controls. Nuclear extracts were isolated (Active Motif, Rixensart, Belgium) and HAT and HDAC activities determined using commercial assays as previously described.^18^

### Cell culture and treatment

HPMECs from healthy subjects (PromoCell GmbH, Heidelberg, Germany) were grown in EGM2 medium and passaged using the Detach subculture kit (PromoCell GmbH) following the manufacturer's instructions. Cells were cultured until 70% confluent at 37°C and 5% CO_2_. Twenty‐four hours prior to experiments, media was changed to EGM2 containing 0.1% foetal calf serum (FCS) before being returned to media containing 5% FCS in the presence or absence of the BET mimic JQ1+ or its inactive enantiomer JQ1−. Cells were used at passages 4–8 and all experiments repeated at least four times.

### Measurement of mRNA transcripts

Total RNA was isolated using RNeasy mini kit (Qiagen, Crawley, UK). Single‐stranded cDNA was synthesized using oligo(dT)_12–18_ primer and MMLV‐reverse transcriptase (Life Technology, Paisley, UK). Quantitative PCR (qPCR) was performed in a Rotor‐Gene 6000 PCR machine (Corbett Research, Cambridge, UK) using a QuantiTect SYBR Green PCR kit and QuantiTect primers (Qiagen). PCR data for each gene was normalized to a housekeeping gene, β‐actin and represented as fold change respective to the t = 0 time point, using the delta–delta CT (2^−∆∆CT^) method.^19^

### Enzyme‐linked immunosorbent assays

CXCL8 (IL8 protein), epidermal growth factor (EGF), fibroblast growth factor (FGF) and ET‐1 in culture supernatants were analysed by sandwich ELISA (R&D Systems, Oxford, UK). IL6 levels were determined by high sensitivity ELISA (eBioscience Ltd, Hatfield, UK).

### ChIP assay

Chromatin immunoprecipitation (ChIP) assay was carried out using EZ‐ChIP kit (Millipore, Watford, UK) according to the manufacturer's protocol. Cells were treated with JQ1 (1 µmol/L) for 3 h and formaldehyde (1% final concentration) was added to fix the protein–DNA complexes. Cells were sonicated to shear the DNA into 100–1000 bp fragments. Immunoprecipitation was carried out overnight with an anti‐p65 antibody (5 µg, Santa Cruz Biotechnology, Santa Cruz, CA, USA). NF‐kB p65 binding to the *IL8* and *IL6* promoter was quantified by real‐time qPCR, using SYBR Green on a Rotor‐Gene 6000 (Corbett Research). The fold change was calculated as (2^−(Ct(input)−Ct(ChIP))^) compared with the IgG negative control. Primer pairs of *IL6* and *IL8* were as follows: *IL6*, forward, 5′‐AGCACTGGCAGCACAAGGCAAAC‐3′ and *IL6*, reverse, 5′‐CAAGCCTGGG‐ATTATGAAGAAGG‐3′; and *IL8*, forward, 5′‐GGGCCATCAGTTGCAAATC‐3′ and *IL8*, reverse, 5′‐TTCCTTCCGGTGGTTTCTTC‐3′.

### Cell viability and proliferation

Cell viability was analysed by quantification of mitochondrial reduction of MTT (3‐(4,5‐dimethyl‐2 thiazolyl)‐2‐5‐diphenyltetrazolium bromide) to formazan. HPMEC proliferation was assessed by measuring the incorporation of bromodeoxyuridine (BrdU) using a cell proliferation ELISA (Roche Diagnostics, Burgess Hill, UK).

### Flow cytometry

FACS analysis was used to measure cell cycle progression. After treatment with JQ1 for 24 h, cells were harvested, spun down at 4°C and washed before being fixed with 70% ethanol. RNA was degraded with RNAase and DNA stained with propidium iodide. Samples were analysed on a BD FACS Canto II (BD Biosciences, Oxford, UK). Histograms were generated and cell cycle analysis was performed using FlowJo cytometry analysis software (FlowJo, LLC, Ashland, OR, USA).

### Western blotting

HPMECs were harvested on ice and proteins isolated for Western blotting as previously described.^20^ Protein content was determined using the Bradford protein assay (Bio‐Rad Laboratories Ltd, Hemel Hempstead, UK). Blots were probed for either cyclin‐dependent kinase (CDK) inhibitor (CDKN) 1A (p21^cip^) or the loading control β‐actin (New England Biolabs (UK) Ltd, Hitchin, UK) and detected using a horseradish peroxidase‐linked anti‐rabbit immunoglobulin (New England Biolabs) and enhanced chemiluminescence (GE Healthcare, Amersham, UK).

### Cell migration

HPMECs were added to the upper chamber of a 24‐well insert (8 mm pore, TranswellH, VWR International Ltd, Lutterworth, Leicestershire, UK) in the presence of JQ1 (1 µmol/L) either added immediately or pre‐incubated for 90 min. Medium (600 μL) containing 0% (negative control) or 5% FCS was added to the lower chamber. After 4 h, cells in the upper chambers were removed with cotton buds. If cells were left for >4 h, they had moved entirely through the membrane and into the lower chamber. Cells on the lower chamber membranes were fixed with 3.7% formaldehyde for 5 min, membrane was then cut and mounted between a slide and a cover slip in ProLong antifade mounting solution containing DAPI (Invitrogen, Paisley, UK). Images were taken using a Zeiss Fluorescence microscope (Carl Zeiss Ltd, Cambridge, UK).

### Statistical analysis

Data are represented as mean ± SEM. Data were analysed by Student's t‐test for two groups of data, by one‐way analysis of variance (ANOVA)/Dunn's multiple comparison test for more than two data sets or by area under the curve (AUC) using GraphPad Prism (GraphPad Prism Software, La Jolla, CA, USA). Differences were considered significant for *P* < 0.05.

## RESULTS

### JQ1 inhibits IL6 and IL8 expression

**Figure 1:**
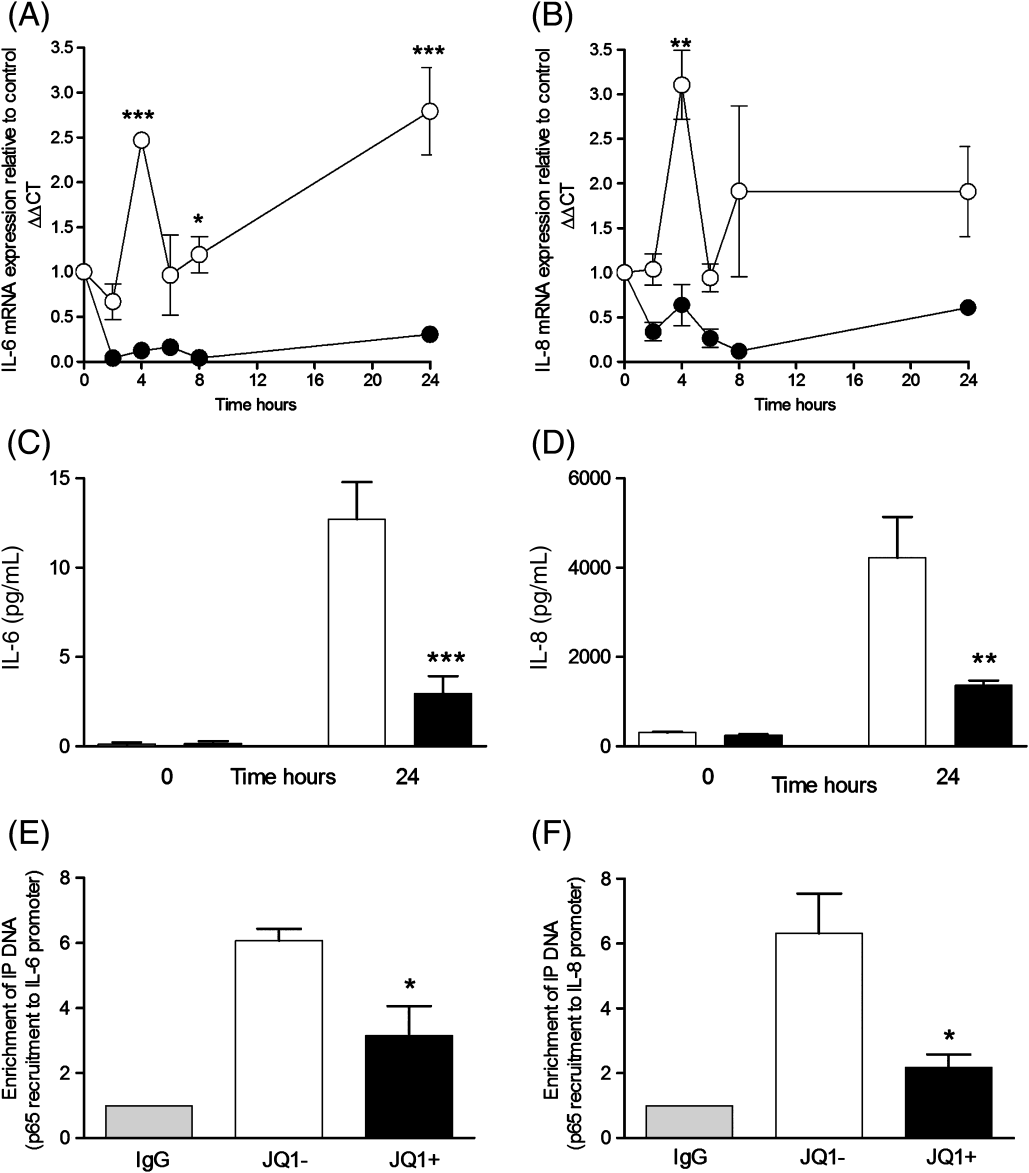
JQ1+ decreases the expression of serum‐stimulated IL6 and IL8 mRNA and protein and decreases recruitment of nuclear factor kappa B (NF‐kB) p65 to IL6 and IL8 promoters in human pulmonary microvascular endothelial cell (HPMEC). Vascular endothelial cells were treated with media (5% foetal calf serum (FCS)) with 1 µmol/L JQ1+ (black circles) or JQ1− (white circles) for 0–24 h. Relative levels of IL6 (A) and IL8 (B) mRNA and IL6 (C) and IL8 protein (CXCL8) (D) release were measured. Chromatin immunoprecipitation (ChIP) analysis of NF‐kB p65 binding to the IL6 (E) and IL8 (F) promoters was quantified by reverse transcription‐quantitative PCR (RT‐qPCR). *P < 0.05, **P < 0.01 and ***P < 0.001 when JQ1+ was compared with JQ1−.

JQ1+ (1 µmol/L) decreased *IL6* mRNA at 4, 8 and 24 h and *IL8* mRNA at 4 h compared with JQ1‐treated cells (Fig. 1A,B). At 24 h, JQ1+ significantly inhibited the release of IL6 and CXCL8 from HPMECs (Fig. 1C,D). JQ1+ had no significant effect on the basal release of EGF, FGF or ET‐1 (data not shown). Cell viability was not affected by JQ1+ or JQ1− at 0–1000 nmol/L.

### JQ1 prevents recruitment of NF‐kB p65 to native IL6 and IL8 promoters

We used ChIP analysis to investigate the effect of JQ1 on the recruitment of NF‐kB p65 to the *IL6* and *IL8* promoters. JQ1+ (1 µmol/L) significantly reduced binding of p65 to the *IL6* promoter by 50% compared with the sixfold enrichment seen in JQ1‐treated cells (Fig. 1E). JQ1+ had a similar effect on p65 recruitment to the *IL8* promoter (Fig. 1F).

### JQ1 inhibits proliferation

**Figure 2:**
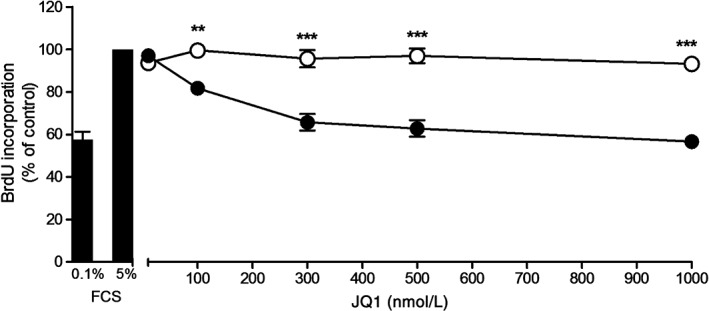
JQ1 decreases serum‐stimulated proliferation in human pulmonary microvascular endothelial cell (HPMEC). Cells were incubated with media containing 0.1% or 5% foetal calf serum (FCS) (Bars) and with 5% FCS media with either JQ1+ (black circles) or JQ1− (white circles) at the stated concentrations for 24 h. Cell proliferation was measured. **P < 0.01 and ***P < 0.001 comparing JQ1+ and JQ1−‐treated cells.

JQ1+ significantly decreased serum‐stimulated proliferation of HPMECs in a concentration‐dependent manner after 24 h. In contrast, there was no effect of JQ1− on serum‐stimulated proliferation (Fig. 2).

### JQ1 prevents cell cycle progression

**Figure 3:**
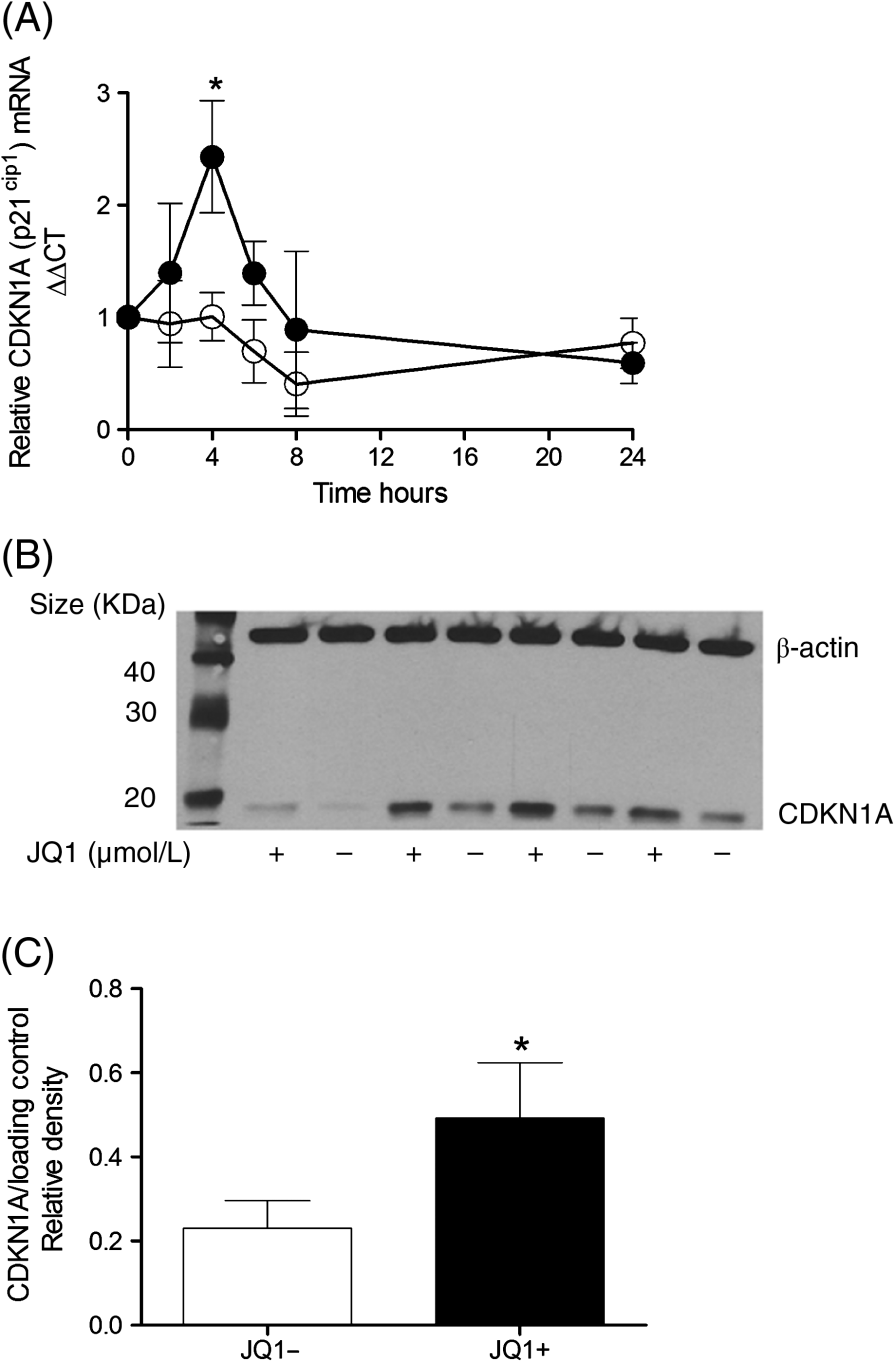
JQ1+ increases cyclin‐dependent kinase inhibitor CDKN1A in human pulmonary microvascular endothelial cell (HPMEC). Vascular endothelial cells were treated with media (5% foetal calf serum (FCS)) containing either 1 µmol/L JQ1+ or JQ1− for (A) 0–24 h and mRNA levels of CDKN1A determined relative to β‐actin by real‐time quantitative PCR (qPCR). (B) A representative Western blot of CDKN1A showing β‐actin as the loading control. (C) Densitometric analysis of n = 4 independent experiments. *P < 0.05 when JQ1+ was compared with JQ1−.

JQ1+, but not JQ1−, significantly increased the percentage of HPMECs in G0/G1 and decreased the percentage of cells in G2/M following serum stimulation (Table 1, Fig. S1 (Supplementary Information)). JQ1+ significantly enhanced CDKN1A (p21^cip^) mRNA expression after 4 h which was not seen with JQ1− (Fig. 3A). The increase in CDKN1A mRNA was mirrored by a significant increase in protein expression at 24 h (Fig. 3B,C).

**Table 1:** Effect of JQ1 on cell cycle progression (flow cytometry analysis) in human pulmonary microvascular endothelial cell (HPMEC)

Concentration		G0/G1 phase	S phase	G2/M phase
10 nmol/L	JQ1^−^	76.27 ± 0.96	3.97 ± 0.37	18.74 ± 0.94
	JQ1^+^	76.43 ± 0.85	4.03 ± 0.33	18.29 ± 0.73
100 nmol/L	JQ1^−^	75.57 ± 1.04	4.23 ± 0.49	18.83 ± 0.80
	JQ1^+^	75.99 ± 1.18	3.76 ± 0.26	18.84 ± 0.88
500 nmol/L	JQ1^−^	76.26 ± 0.91	3.88 ± 0.26	18.57 ± 0.91
	JQ1^+^	78.84 ± 1.58	2.57 ± 0.35	17.10 ± 1.05
1000 nmol/L	JQ1^−^	75.74 ± 1.05	4.05 ± 0.33	19.09 ± 1.09
	JQ1^+^	82.14 ± 0.86***	1.38 ± 0.05	15.41 ± 0.88*

Cells were treated with 0‐1000nmol/L JQ1(+) or JQ1(−) in full media for 24 h. Samples were analysed on a BD FACS Canto II and histograms generated and cell cycle analysis performed using FlowJo. Data are mean ± SEM, *n* = 6, *P < 0.05, ***P < 0.001, when compared with the same concentration of inactive JQ1.

**Figure 4:**
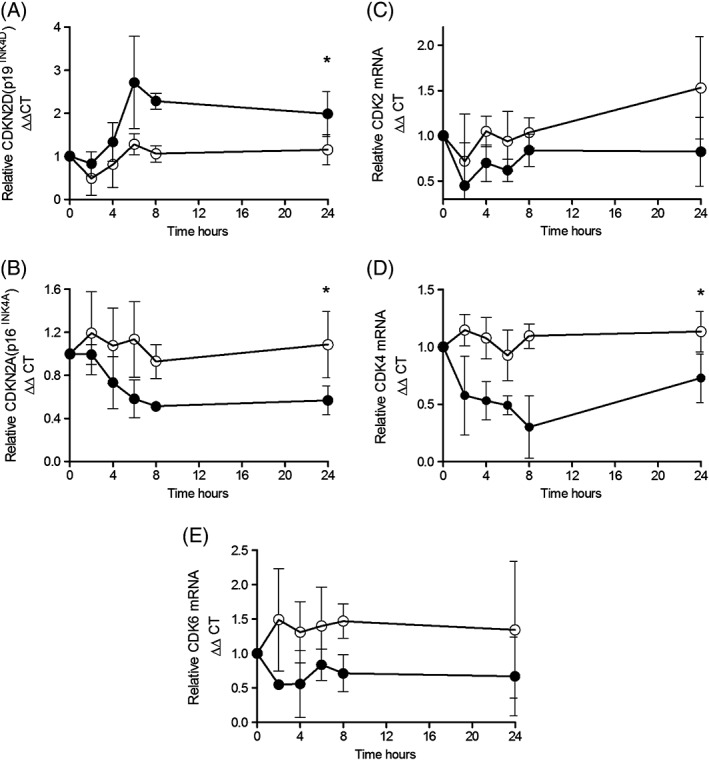
Effect of JQ1 on human pulmonary microvascular endothelial cell (HPMEC) cell cycle genes involved in G1 to S phase progression. Cells were treated with media (5% foetal calf serum (FCS)) containing either 1 µmol/L JQ1+ (black circles) or JQ1− (white circles) for 0–24 h and relative mRNA levels of cyclin‐dependent kinase (CDK) inhibitors (A,B) and CDKs (C–E) are shown. *P < 0.008 when the area under the curve for JQ1+ was compared with JQ1−.

JQ1+ also significantly increased CDKN2D (p19^INK4D^) mRNA expression compared to JQ1− (Fig. 4A) and significantly decreased CDKN2A (p16^INK4A^) (Fig. 4B). No change in the mRNA levels of CDKN1B (p27^kip1^) or CDKN2B (p15^INK4B^) was observed (Fig. S2, Supplementary Information). JQ1+ also reduced CDK2, CDK4 and CDK6 mRNA levels compared with cells treated with JQ1− but this only reached significance for CDK4 (Fig. 4C–E).

### JQ1+ inhibits HPMEC migration

**Figure 5:**
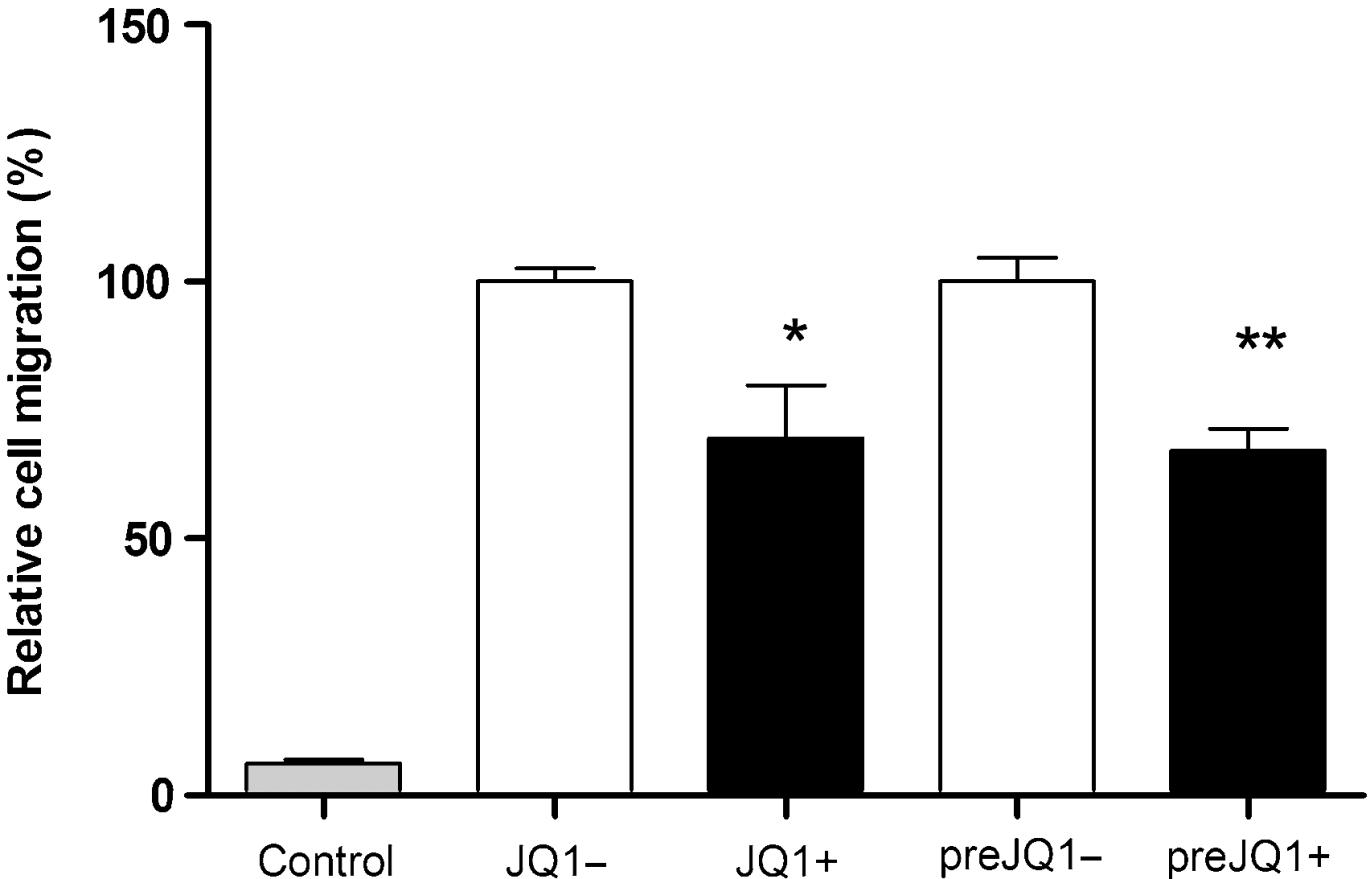
JQ1+ decreases serum‐stimulated migration of human pulmonary microvascular endothelial cell (HPMEC). HPMECs were seeded onto transwell inserts and cell migration was measured. Migrated cells, under the conditions described, were counted and expressed graphically, as the percentage of the control. Data are presented as mean ± SEM. *P < 0.05 and **P < 0.01.

JQ1+ (1 µmol/L) caused a significant decrease in serum‐stimulated endothelial cell migration compared with JQ1− after 4 h (Figs 5, S3 (Supplementary Information)). Pre‐treatment of HPMECs with JQ1+ produced a similar level of suppression of migration compared with that seen when JQ1+ was added at the same time as the stimulus.

### Disrupted HAT activity in IPAH lung

**Figure 6:**
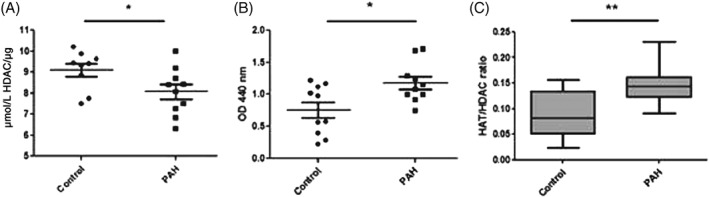
Histone acetyltransferase (HAT) and histone deacetylase (HDAC) activity in lung tissue from idiopathic pulmonary arterial hypertension (IPAH) and controls. Nuclear extracts were prepared from whole lung tissue from IPAH (n = 10) and controls (n = 10) and HDAC (A) and HAT (B) activity and their ratio were determined (C). *P < 0.05, **P < 0.01.

Lung tissue from IPAH patients showed significantly decreased nuclear HDAC and increased nuclear HAT activity compared with non‐PAH lung tissue (Fig. 6A,B). This resulted in a higher HAT:HDAC ratio in IPAH lung tissue compared with non‐PAH tissue samples (Fig. 6C).

## DISCUSSION

The BET mimetic JQ1+, but not its inactive enantiomer, JQ1−, decreased IL6 and IL8 mRNA and protein expression from HPMECs, which was associated with reduced p65 recruitment to the native *IL6* and *IL8* promoters. JQ1+ also decreased serum‐stimulated proliferation of HPMECs. JQ1+ caused cell cycle arrest at G1 linked to increased expression of the cell cycle inhibitors CDKN1A (p21^cip^) and CDKN2D (p19^INK4D^) together with reduced CDK2, CDK4 and CDK6 expression. Furthermore, JQ1+ inhibited serum‐stimulated migration of HPMECs. Finally, we demonstrated that there is increased HAT and reduced HDAC activity in IPAH lung.

The pulmonary endothelium controls vasodilator/constrictor balance as well as vascular permeability and regulates the local balance between pro‐ and anti‐inflammatory mediators, anti‐coagulant and anti‐adhesive properties and cell proliferation.^21^ Vasomotor tone is controlled by the balance between vasodilators such as nitric oxide and prostacyclin and vasoconstrictors such as ET‐1.^21^ This balance is lost in PAH patients with increased ET‐1 release and reduced nitric oxide and prostacyclin production and current therapies are aimed at countering this imbalance. However, these therapies do not treat the other hallmark of PAH; pulmonary vascular remodelling.

A key event in PAH is NF‐kB activation within the vascular endothelium of IPAH lung. These endothelial cells release NF‐kB‐regulated cytokines and chemokines, including IL6, IL8 and MCP‐1 whose expression are increased in the plasma of patients with IPAH and is predictive of clinical outcome.^5^, ^22^ Novel agents are required that target these abnormalities. The only drug to date to target remodelling *per se* is the PDGF receptor kinase inhibitor, imatanib, which reduced pulmonary vascular resistance in patients with severe PAH but its use was associated with intolerable side effects.^23^, ^24^ We show that the BET mimetic, JQ1+ (which has a higher affinity for BRD4 than other Brds^25^), inhibits key inflammatory processes in human primary HPMECs. Further studies in patient cells will determine which BET proteins may be important in PAH pathogenesis.

Serum stimulates HPMEC proliferation through multiple potential pathways, including, the production of IL6 and causes endothelial cell migration.^26^ In this study, we show for the first time that JQ1+ inhibits serum‐stimulated proliferation and migration of HPMECs. Furthermore, we demonstrated that JQ1 increased CDKN1A and CDKN2D levels which was associated with increased G0/G1 arrest. Interestingly, CDKN1A levels were decreased in a rodent model of PAH^27^ and JQ1 increased CDKN1A protein expression in PAH‐PASMCs within 16 h of treatment showing rapid arrest of proliferation by BRD4 inhibition.^11^ Previous studies have shown that JQ1 inhibited proliferation and differentiation of mouse cancers in vivo and of a number of other cell lines in vitro.^17^ This suggests a potential anti‐remodelling effect of JQ1+ by BRD4 inhibition in pulmonary microvascular endothelial cells.

In this study, we only used serum as the stimulus for cytokine production and in future studies it would be important to investigate whether JQ1+ can attenuate proliferation and remodelling induced by other stimuli such as shear stress, hypoxia and cytokines as well as comparing responses to cells derived from patients with PAH and in other cell types such as smooth muscle cells and fibroblasts. The effect of JQ1+ in an animal model of PAH may further elucidate the potential of this class of drugs for the treatment of the disease.

The HAT:HDAC activity ratio was significantly altered in whole lung tissue of IPAH patients. Although we do not provide evidence of cellular specificity, it does suggest that altered acetylated histone states could be important in the development of human PAH. The relative protein or mRNA expression of BET isoforms in HPMECs in healthy patients or in disease is unknown. However, BRD4 is increased in PAH lung tissue, distal PAs, RV and isolated PASMCs compared with control tissues and cells. Levels of BRD2 and BRD3 are increased in the distal PAs from PAH patients compared with controls, although the difference in BRD3 levels was not significant. In the RV, no increase in BRD2 and only a small increase in BRD3 were observed.^11^ Enhanced BRD4 expression was also induced during RV hypertrophy in a rat PAH model.^12^ BRD4 mRNA is expressed at 10 times the level of BRD2 and BRD3 in cardiomyocytes and prophylactic JQ1 treatment is effective at counteracting the effect of two different inducers of left ventricular heart failure particularly in relation to the inflammatory drive.^28^ This provides an excellent rationale for the extension into therapeutic interventions in a mouse model of PAH. Indeed, non‐specific histone acetylation modifiers reverse the development of PAH and both right and left ventricular function in several animal models.^29^, ^30^, ^31^

There are a number of BRD proteins that perform cell‐specific roles. BRD2 has an anti‐inflammatory role in murine macrophages by targeting acetylated p65,^14^ whereas BRD4, but not BRD2, has an anti‐inflammatory effect in primary human airway structural cells independent of p65 acetylation.^32^ Our study does not define the precise mechanism or role of individual BET proteins in regulating inflammation in pulmonary vascular endothelial cells. However, we have shown that the BET mimic JQ1+ has potentially important anti‐inflammatory and anti‐remodelling effects on HPMECs which could be of relevance to the pathogenesis of PAH. Further work using JQ1+, or similar drugs, is necessary in cells from PAH patients and in relevant in vivo models.

## Supporting information


**Figure S1** JQ1+ decreases cell cycle progression.
**Figure S2** Effect of JQ1 on human pulmonary microvascular endothelial cell (HPMEC) cycle genes involved in G1 to S phase progression.
**Figure S3** JQ1+ decreases serum‐stimulated migration of human pulmonary microvascular endothelial cell (HPMEC). HPMECs were seeded onto transwell inserts and cell migration measured. Representative images of cells from three independent experiments (nuclei identified with DAPI staining) migrated to the lower chamber are shown.
