# Supplementary material for: Bromodomain and extra‐terminal protein mimic JQ1 decreases inflammation in human vascular endothelial cells: Implications for pulmonary arterial hypertension
Source: Respirology. 2016 Aug 18;22(1):157–64. doi: 10.1111/resp.12872 (PMC5215513; doi:10.1111/resp.12872)
Supplement: Supplementary file 1 — Figure S1 JQ1+ decreases cell cycle progression. Figure S2 Effect of JQ1 on human pulmonary microvascular endothelial cell (HPMEC) cycle genes involved in G1 to S phase progression. Figure S3 JQ1+ decreases serum‐stimulated migration of human pulmonary microvascular endothelial cell (HPMEC). HPMECs were seeded onto transwell inserts and cell migration measured. Representative images of cells from three independent experiments (nuclei identified with DAPI staining) migrated to the lower chamber are shown. [file RESP-22-157-s001.docx]

**SUPPLEMENTARY INFORMATION**

**Bromodomain and extra-terminal (BET) protein mimic JQ1 decreases inflammation in human vascular endothelial cells: Implications for pulmonary arterial hypertension.**

Sharon Mumby,^ab^ Natalia Gambaryan,^a^ Chao Meng,^a^ Frederic Perros,^c,d^ Marc Humbert,^c,d,e^ S. John Wort,^a^ Ian M. Adcock ^b^

^a^Vascular Biology and ^b^Airway Disease Section, National Heart and Lung Institute, Imperial College London, London, United Kingdom. ^c^Université Paris-Sud, Faculté de Médecine, Kremlin-Bicêtre, France. ^d^Institut National de la Santé et de la Recherche Médicale U999, Hypertension Artérielle Pulmonaire: Physiopathologie et Innovation Thérapeutique, Le Plessis-Robinson, France. ^e^Assistance Publique-Hôpitaux de Paris, Centre National de Référence de l'Hypertension Pulmonaire Sévère, Service de Pneumologie et Réanimation Respiratoire, Hôpital Antoine Béclère, Clamart, France.

**Figure S1- JQ1+ decreases cell cycle progression**


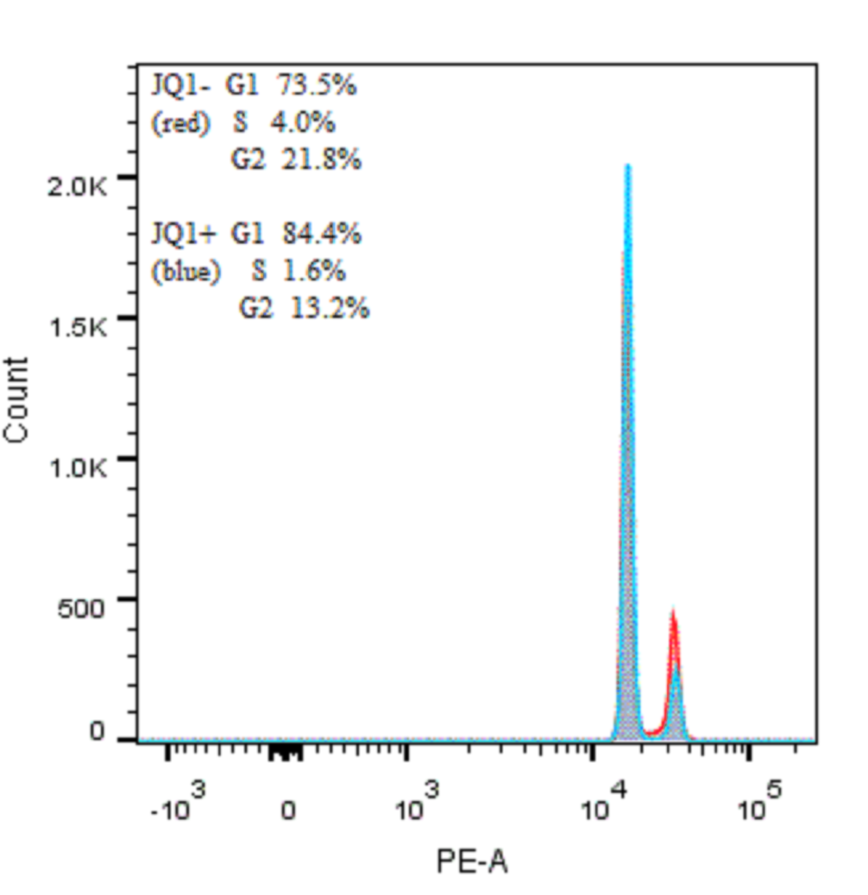


Representative histogram to show percentage of cells in the G1, S and G2 phases of the cell cycle after treatment with 1µM JQ1+ (blue) and JQ1- (red) as measured by Flow Cytometry

**Figure 2: Effect of JQ1 on HPMEC cell cycle genes involved in G1 to S phase progression**


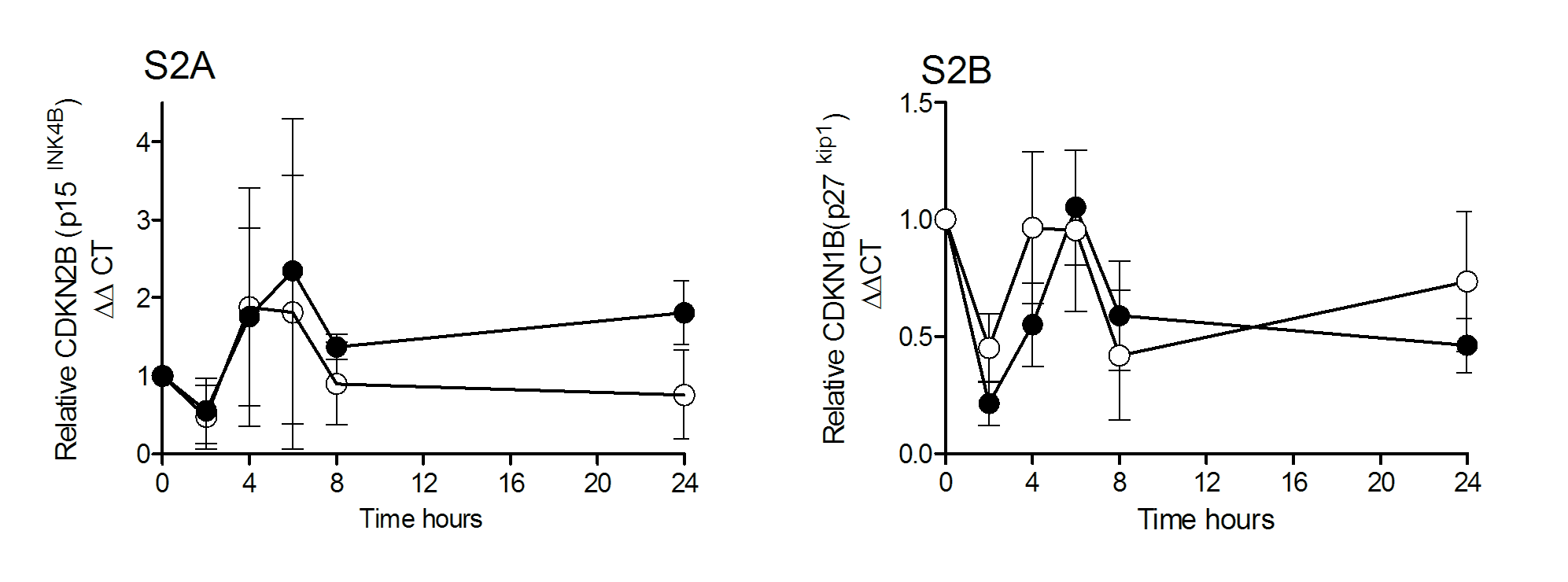


**.** Cells were treated with media (5% FCS) containing either 1µM JQ1+ (black circles) or JQ1- (white circles) for 0-24 hours and relative mRNA levels of (A_ CDKN2B (p15^INK4B^) and (B) CDKN1B (p27^kip1^) are shown.

**Figure 3: JQ1+ decreases serum-stimulated migration of HPMEC.**


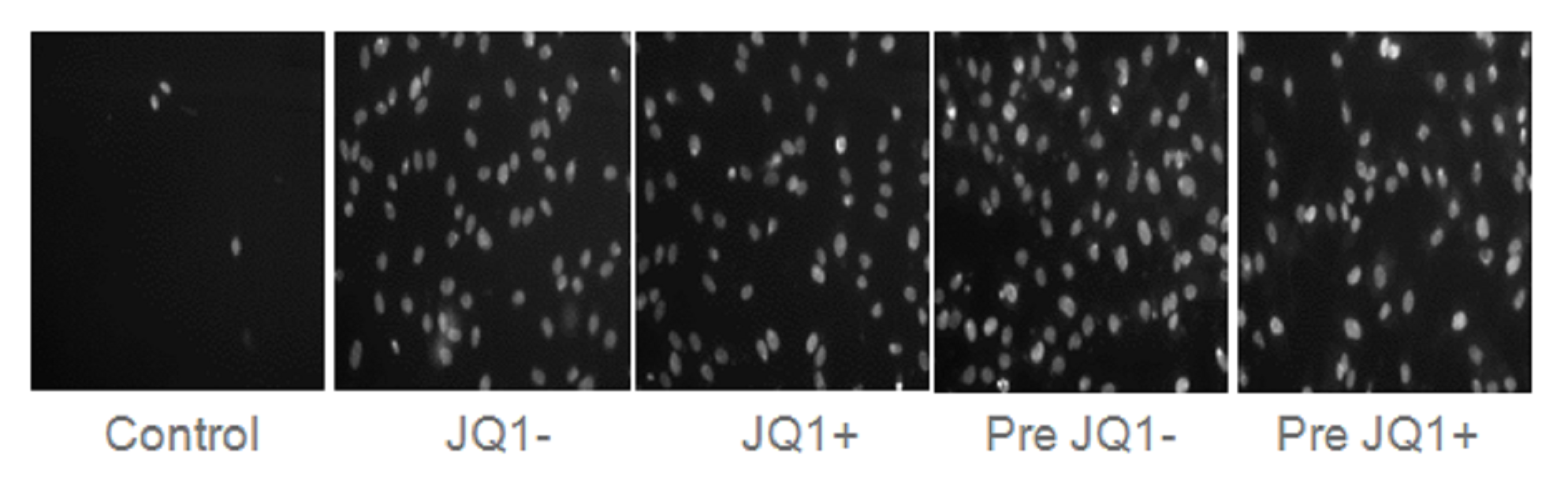


HPMECs were seeded onto transwell inserts and cell migration measured. Representative images of cells from 3 independent experiments (nuclei identified with DAPI staining) migrated to the lower chamber are shown.
